# Too “sexy” for the field? Paired measures of laboratory and semi-field performance highlight variability in the apparent mating fitness of *Aedes aegypti* transgenic strains

**DOI:** 10.1186/s13071-019-3617-2

**Published:** 2019-07-19

**Authors:** Andrew Aldersley, Arissara Pongsiri, Kamonchanok Bunmee, Udom Kijchalao, Wachiraphan Chittham, Thanyalak Fansiri, Nattaphol Pathawong, Alima Qureshi, Laura C. Harrington, Alongkot Ponlawat, Lauren J. Cator

**Affiliations:** 1 Department of Life Sciences, Imperial College London, Silwood Park, Ascot, UK; 2 Vector Biology and Control Section, Department of Entomology, Armed Forces Research Institute of Medical Sciences, Bangkok, Thailand; 3 Department of Medical Entomology, Faculty of Tropical Medicine, Mahidol University, Bangkok, Thailand; 4 Department of Entomology, Cornell University, Ithaca, NY USA

**Keywords:** *Aedes aegypti*, Mating success, Vector control, Body size, Larval conditioning

## Abstract

**Background:**

Evaluating and improving mating success and competitive ability of laboratory-reared transgenic mosquito strains will enhance the effectiveness of proposed disease-control strategies that involve deployment of transgenic strains. Two components of the mosquito rearing process, larval diet quantity and aquatic environment - which are linked to physiological and behavioural differences in adults - are both relatively easy to manipulate. In mosquitoes, as for many other arthropod species, the quality of the juvenile habitat is strongly associated with adult fitness characteristics, such as longevity and fecundity. However, the influence of larval conditioning on mating performance is poorly understood. Here, we investigated the combined effects of larval diet amount and environmental water source on adult male mating success in a genetically modified strain of *Aedes aegypti* mosquitoes in competition with wild-type conspecifics. Importantly, this research was conducted in a field setting using low generation laboratory and wild-type lines.

**Results:**

By controlling larval diet (high and low) and rearing water source (field-collected and laboratory water), we generated four treatment lines of a genetically modified strain of *Ae. aegypti* tagged with fluorescent sperm. Laboratory reared mosquitoes were then competed against a low generation wild-type colony in a series of laboratory and semi-field mating experiments. While neither food quantity nor larval aquatic environment were found to affect male mating fitness, the transgenic lines consistently outperformed wild-types in laboratory competition assays, an advantage that was not conferred to semi-field tests.

**Conclusions:**

Using a model transgenic system, our results indicate that differences in the experimental conditions of laboratory- and field-based measures of mating success can lead to variation in the perceived performance ability of modified strains if they are only tested in certain environments. While there are many potential sources of variation between laboratory and field lines, laboratory adaptation - which may occur over relatively few generations in this species - may directly impact mating ability depending on the context in which it is measured. We suggest that colony-hybridization with field material can potentially be used to mitigate these effects in a field setting. Release programs utilising mass-produced modified laboratory strains should incorporate comparative assessments of quality in candidate lines.

**Electronic supplementary material:**

The online version of this article (10.1186/s13071-019-3617-2) contains supplementary material, which is available to authorized users.

## Background

*Aedes aegypti* is the primary vector of a range of arboviruses including dengue, chikungunya, Zika, and yellow fever, that affect the health of millions of people annually [1, 2]. Recent efforts to control the spread of these diseases have focussed on the release of modified individuals to limit the reproductive or transmission capacity of local mosquito populations [3, 4]. Several such programs have now been extensively trialled [5–8]. In general, these strategies require released males to mate with wild females in the selected area to introduce a replacement or limiting mechanism into the target population [9]. The fitness of release lines is thus vital to program success; modified males not only must survive and disperse within the target environment, but also compete with wild conspecifics for mates [10–13]. One challenge to optimising male performance is understanding what key fitness parameters should be measured in the laboratory and how they relate to male fitness in the field [14]. Understanding and improving the performance of release males therefore has the potential to influence the operational success or failure of vector management initiatives.

All mosquito species are aquatic as juveniles [15]. Development of *Ae. aegypti* typically takes place in water-filled containers found among households in urban and rural areas, such as discarded tyres, flower pots and buckets [16]. Larvae obtain food and nutrients from their surrounding habitat, feeding on plant matter and other organically-derived detritus [17]. Both the amount and content of food available affect larval development in *Ae. aegypti*. Variability in the quality of natural container environments is often high, resulting in heterogeneity in the physiological, nutritional, and energy status of the adult population [18–20].

As in many other invertebrate systems [21], larval conditioning has been linked to adult fitness in *Ae. aegypti*. Larval food quantity is one of the major determinants of adult body size [22], which is associated with a range of life history traits. Larger females are both more fecund and have a greater longevity [23, 24], while male size is positively correlated with sperm production and transfer [25, 26], survival [27], and increased pre-copulatory flight activity [28]. As larvae, mosquitoes also acquire a diverse microbiome from the rearing environment, which influences their development [29, 30] and adult physiology [31]. In addition, exposure to different microbes at the juvenile stage has recently been demonstrated to affect both body size [32] and egg production [33] in *Ae. aegypti.* Nutritional and microbial conditions experienced by larvae are known to interact with factors that drive vector competence in several medically important mosquito species [34–42]. In many insects, the microbiome governs adult behavioural processes [43]. However, the effect of the larval environment on adult mating competitiveness, a key aspect of mosquito fitness [10], is less understood.

This consideration is particularly relevant for those developing transgenic mosquito lines destined for release. The relationship between size and mating success in *Aedes* is unclear, with some studies indicating that small females preferentially mate with small males [44], while others have reported high copulation rates in large females regardless of male size [26, 45]. In contrast to field-bred individuals, laboratory lines are often sustained under conditions intended to optimise developmental time and synchrony [44], generating cohorts that tend to be larger, and with a lower size variance, than wild populations [46]. Similarly, the role of the microbiome is often overlooked during production of release strains. Evidence from varied dipterans indicates that changes to the host microbiota can alter the sexual competitiveness [47–49] or mating preferences [50–52] of laboratory lines. These effects may be compounded by selective pressures introduced by prolonged laboratory rearing [53], which can result in habituation and reduced fitness in the field [54, 55]. Thus far, studies into the mating success of transgenic or laboratory-reared *Ae. aegypti* have analysed factors such as male insemination capacity [25, 26, 56], pre-copulatory signalling ability [45], and female fecundity [23, 57]. Investigations of mating competitiveness in this species are limited. Most have applied laboratory-based assays [28, 58, 59], while those utilising field approaches are relatively few [60, 61]. It is not currently clear which quality control measures are most important for evaluating performance in mosquito release lines, yet larger-scale trials in natural conditions are considered an important tool in this process [55, 62, 63].

Here, we tested the effects of larval diet amount and rearing water type on adult male mating success in a genetically modified laboratory strain of *Ae. aegypti*. We used an integrated approach combining laboratory and semi-field assays to assess several aspects of male mating fitness in competition with wild-type conspecifics in Kamphaeng Phet (KPP), Thailand: longevity, attendance to swarm sites, copulation and insemination success. We predicted that laboratory-sourced males fed on high-quantity diets supplemented with water from naturally productive larval environments would enjoy a greater mating success against wild-type males than those fed on low-quantity diets or reared in laboratory water. We did not find evidence to support this prediction; male performance was not improved by increasing diet amount or by supplementing rearing environments with field-container water. However, males from the laboratory reared lines were found to outcompete wild-type conspecifics in laboratory mating assays. Importantly, laboratory-reared males did not display this advantage in semi-field cage trials. Our results suggest that adaptation to laboratory mating conditions can inflate the perceived mating success of release lines if they are only measured under laboratory conditions.

## Methods

### Description of field site

All experiments were conducted in the dengue endemic area of Muang District, Kamphaeng Phet (KPP) Province, Thailand, which is located approximately 360 km northeast of Bangkok. In 2018, there were 917 dengue cases and 2 deaths reported to the Ministry of Public Health in this region [64]. All four dengue serotypes have circulated in KPP [65]. The climate is tropical with three seasons: rainy (mid-May to mid-October), winter (mid-October to February), and summer (mid-February to mid-May) [66]. Investigations were performed at the Large Mosquito Enclosure semi-field facility constructed by the Department of Entomology, Armed Forces Research Institute of Medical Sciences (AFRIMS), Thailand in September and October 2018.

**Fig. 1:**
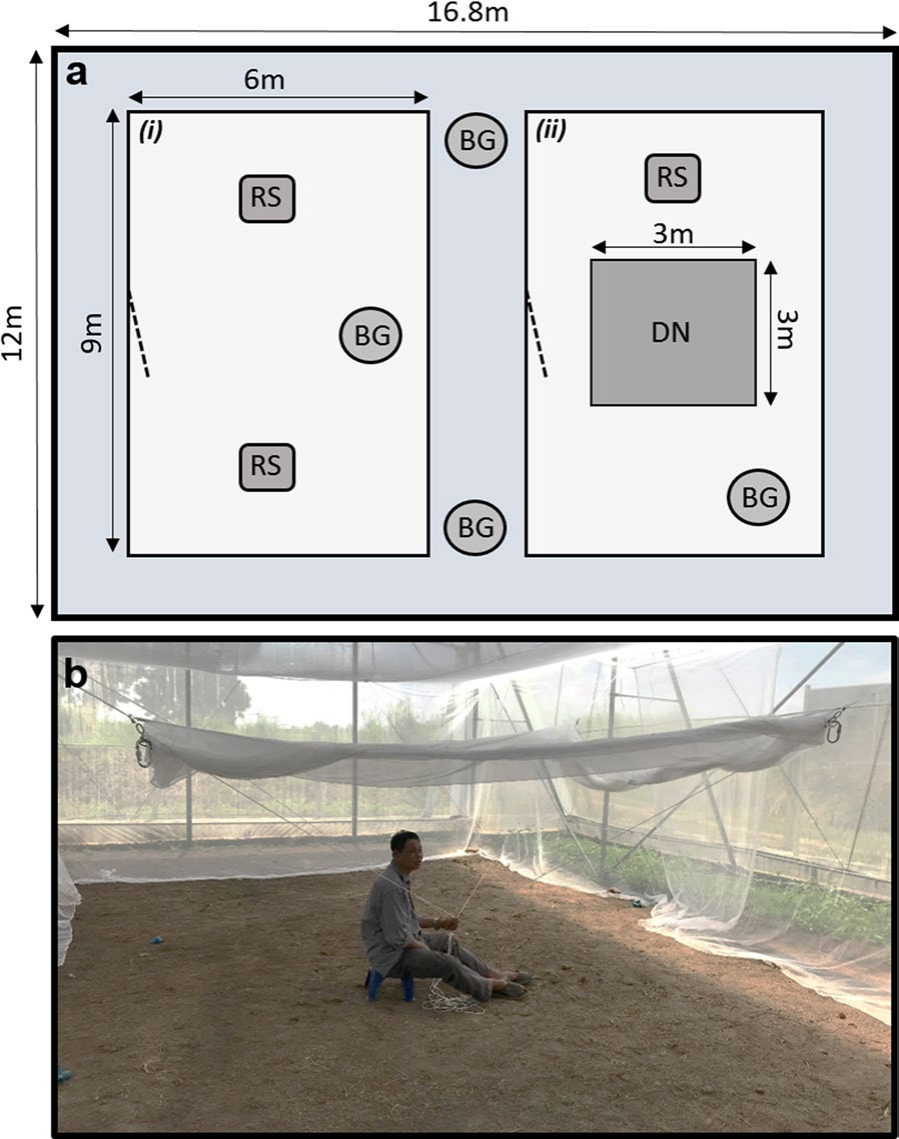
Layout of individual semi-field cages. **a** Two sub-enclosures constructed from bednet material were erected inside each semi-field cage structure. These had entrance points on the left-side (dashed line). The leftmost sub-enclosure (*i*) was used for mating competition assays, while the right (*ii*) was used for swarm site attendance experiments. In (*ii*), a drop-net (DN) was hung and used to capture males attracted to the host. BG-Sentinel traps (BG) were left inside the sub-enclosures and in the main semi-field cage area for mosquito recapture. Resting sites (RS), partially filled with water, were also placed inside each experimental arena. Access to the semi-field cages was controlled by a double sliding door. **b** Drop-net procedure for measuring swarm site attendance inside a semi-field cage sub-enclosure. Host is positioned under the raised drop-net, which is released after 10 min. Male mosquitoes in close proximity to the host are trapped and recaptured using a vacuum aspirator

The AFRIMS site consists of three semi-field cages, 16.8 × 125.5 m metal structures with a covered roof, semi-permeable mesh outer-walls, and dirt floors with sparse vegetation. The semi-field cages provide an enclosed space with a similar climatic profile to the natural habitat of *Ae. aegypti*. These outdoor facilities were observed to contain a number of common mosquito predators (geckos, spiders and ants). Within each semi-field cage, we erected two further 6 × 9 × 3 m secondary containment areas constructed from bed-net material (Fig. 1a). These sub-enclosures were used to conduct semi-field mating competition and swarm site attendance, which are described in the sections detail below. Two BG-Sentinel traps (Biogents GmbH, Regensburg, Germany) baited with dry-ice and BG-Lure cartridge human odour were placed inside each semi-field cage in the area outside the sub-enclosures. These were left running constantly and monitored daily during experiments to estimate the escape-rate from the secondary containment cages. Mosquitoes captured in these traps were not included in analyses. All other experimental work was performed in adjacent temperature-controlled laboratory buildings on site. Climatic conditions were monitored in all rearing environments, laboratories, and the semi-field cages using a combination of EasyLog USB (Lascar Electronics Ltd, Whiteparish, Wiltshire, UK) and HOBO (Onset Computer Corporation, Bourne, MA, USA) data loggers. Temperatures averaged 30.7 ± 5.3 °C in semi-field cages over the duration of the test period, with a relative humidity of 74.5 ± 17.6%.

### Survey and collection from natural habitats

We surveyed domestic sites in KPP for active water storage containers (sampling dates: 10th, 16th and 17th September, and 1st and 2nd October 2018). A container was considered to be active when at least 10 fourth-instar *Aedes* mosquito larvae and/or pupae (identified using physiological indicators [67]) were easily observed inside it. We collected water (a minimum of 0.5 l), debris, larvae, and pupae from a total of 21 containers. Samples were strained of large organic matter and living organisms and combined into a field-container mixture used during rearing of experimental lines (see below). This was supplemented with rainwater collected at the field laboratory site when necessary. We used this homogenised blend of container samples to represent the microbial community present in natural breeding sites of *Ae. aegypti* in KPP.

### Mosquito lines and rearing

A laboratory strain of *Ae. aegypti* mosquitoes homozygous for the fluorescent protein DsRed [68] was produced from a cross between transgenic Higgs’ White Eye (HWE) DsRed mosquitoes and a third generation field line originating from KPP (DsRedKPP line, details in Additional file 1: Text S1). We subjected this line to treatment conditions that manipulated both the larval rearing water type (laboratory or field-container water) and the quantity of food they were offered (Cichlid Gold, Hikari, Kyrin Food Industries Ltd, Japan): high (1.0 mg/larvae/day) or low (0.3 mg/larvae/day ground fish food); details of the diet selection process are given in Additional file 1: Text S2. This regime yielded adults from four different diet-water treatment combinations: High-Lab (HL); Low-Lab (LL); High-Field (HF); and Low-Field (LF). DsRedKPP eggs were submerged in mains-supplied tap water from the field laboratory and left for a maximum of 24 h to induce hatching. First-instar larvae were separated into plastic trays (33 × 23 × 9 cm) containing 2 l of water and reared at a density of 250 larvae/l. Larvae from the HL and LL treatments were reared in laboratory tap water (as above). In the HF and LF treatments, larval trays were filled with 1.6 l of laboratory tap water and 400 ml of the field-container mixture. Trays were covered with netting and stored under ambient conditions (29.6 ± 2.9 °C and 76.0 ± 11.5% relative humidity over the duration of the rearing period) on shelving units inside a small windowed building.

A competitor line of KPP wild-type *Ae. aegypti* (KPPWT line) was established by sampling larvae (*c*.2000) from villages in 3 different subdistricts of KPP in July 2018. These larvae were mixed in trays (as described above) filled with field-container water and allowed to eclose. Adults were sorted to retain only *Ae. aegypti*, which were mixed in cages (30 × 30 × 30 cm), offered a 10% sucrose solution, and permitted to mate. Females (*c.*800) were given a human blood meal and allowed to oviposit, from which we obtained first generation offspring. These individuals were hatched in large black plastic containers (44 × 34 × 17 cm) filled with a mixture of laboratory tap water (14 l), rainwater collected from the field laboratory site (1.6 l), and the field-container mixture (400 ml). Trays contained approximately 1000 larvae. On each day, 400 ml of water was decanted from the rearing trays and replaced with a fresh sample of the field-container mixture. From the fourth day after hatching, we supplemented the available nutrients in the trays daily with 6 mg of ground fish food. The KPPWT trays were also covered with netting and stored outside in the shade (29.3 ± 3.8 °C and 29.7 ± 8.7% relative humidity over the duration of the rearing period).

Upon pupation, individuals from each treatment were transferred into individual tubes. Newly eclosed adult males and females were placed into separate cages (30 × 30 × 30 cm) for each treatment condition and offered a 10% sucrose solution. All adult cages were stored in a temperature-controlled laboratory building 28.4 ± 1.5 °C and 28.3 ± 4.2% relative humidity over the course of the test period). To monitor and control adult ages throughout experiments, new cages were established every 2 days.

### Nutritional analyses

We conducted nutritional analyses (MR4 protocol adapted from [69–72]) on a subsample of freshly eclosed males from the four DsRedKPP treatments (LL, LF, HL and HF) and the KPPWT line used in experiments. In addition, we also ran analyses on pupae collected from six of the productive containers identified during the field survey. Sugar, lipid, and glycogen stores for each male were quantified using a colorimetric method.

### Experimental design

We performed a minimum of two replicates for each of the assays described below. These replicates were conducted in three blocks due to the availability of three semi-field cages at the field laboratory site. Blocks were staggered such that they successively overlapped, beginning every 4–5 days (full details of the treatments included in each block are given in Additional file 2: Table S2). KPPWT mosquitoes were hatched periodically every 3 days to ensure a regular supply of appropriately-aged adults was available for each blocked experiment. To prevent cross-contamination across blocks, we ensured a break of at least 24 h between experiments conducted inside the semi-field cages. Furthermore, adults from the different treatments were rotated across all semi-field cages both between (mating competition assay) and within (swarm site attendance assay) blocks, to account for any variation in semi-field cage microenvironments that could have affected experimental outcomes.

### Adult survival assay

We recorded the adult survival of a subset of individuals from the High-Lab (HL), Low-Lab (LL) KPPWT lines. We took 10 males from each treatment (less than 1-day from eclosion) and placed them together into a small cup (0.45 l). These were then offered either a 10% sucrose solution (*n* = 2 containers per treatment) or water only (*n* = 5 containers per treatment) soaked into cotton wool. Adults were held inside under ambient conditions (30.3 ± 2.4 °C and 69.8 ± 7.5% relative humidity). Mortality was monitored in each container daily.

### Laboratory mating competition

We investigated the impact of larval diet on male mating response and success when in direct competition with wild-type individuals. Following previous methodology [28], two 2–6 day-old virgin males from one of the four diet-water treatments (DsRedKPP) were competed against two 2–4 day-old virgin KPPWT males for a single 2–4 day-old virgin wild-type female (KPPWT). A subset of males from each treatment condition was selected at random and marked with either yellow (DsRedKPP strains) or pink (KPPWT strain) dust (Swada Inc, Stalybridge, Cheshire, UK) [28, 73], then left overnight to recover. The following day, males were aspirated into a cylindrical container (2.83 l) and left for 5 min to acclimate. A single KPPWT female was then introduced into the cage. Host cues were provided by proximity of the investigator. The individuals were left undisturbed under observation until formation of the first copula (defined as genital contact for a minimum of 5 s), at which point the mating pair was aspirated from the cage (whilst still coupled). The strain of the successful male (DsRedKPP or KPPWT) was identified by inspection of dust colour. This assay was repeated 20 times in two replicates for each diet-water treatment. The right wings of the females and successful and unsuccessful males from these experiments were dissected and measured to estimate body sizes [74] across the treatment groups.

### Semi-field cage mating competition assay

We measured the effect of larval rearing treatment on male mating success under semi-field conditions. To begin, 100 2–5 day-old virgin KPPWT females were released into one of the semi-field cage sub-enclosures (Fig. 1a) between 7:00 and 7:30 h, and allowed to naturally disperse and settle. At either end of the sub-enclosure were positioned two resting sites consisting of an open-topped black-cloth covered box (35 × 35 × 55 cm) with a partially-filled bucket of water placed inside (Fig. 1a). Approximately 60 min later, 100 3–7 day-old virgin DsRedKPP males taken from one of the four larval treatments were released into the sub-enclosure, along with 100 2–5 day-old virgin KPPWT males. Males were released simultaneously from separate containers placed next to one another at the rear-end of the sub-enclosure. Mating was allowed to occur in the enclosure for 10–11 hours. Host stimuli were provided by walking around the edges of the cages in the morning and late-afternoon of the release day. Starting between 18:00 and 18:30 h, surviving individuals were collected back using a BG-Sentinel trap located inside the sub-enclosure (Fig. 1a). The following morning and evening, we entered the sub-enclosures and collected any remaining mosquitoes using a prokopack [75] or CDC vacuum aspirator. The BG-Sentinel trap was left running for 36 h and checked twice-daily to maximise mosquito recapture.

Collected females were immediately sedated with cold and dissected in PBS to determine their mating status. The presence of sperm in the spermathecae confirmed successful insemination. We used a DsRed filter attachment (ET-CY3/TRITC, Chroma Technology Corporation, Vermont, USA) on a stereomicroscope (BX50 Fluorescence Microscope, Olympus Corporation, Tokyo, Japan) to visualise florescent sperm in the spermathecae and infer whether the female was mated by a male from the DsRedKPP (positive DsRed signal) or KPPWT (negative DsRed signal) strain. Mating success was defined as the proportion of recaptured females that were successfully inseminated by released males from either respective treatment. We also counted the number of recaptured males to provide an overall estimate of mortality rates across the semi-field cages.

### Swarm site attendance

We tested the effect of larval rearing condition on adult male attendance at swarm sites inside the semi-field environment. Ten 2–6 day-old virgin male KPPWT and DsRedKPP mosquitoes (from one of the four diet-water treatments) were dusted with either pink (KPPWT) or yellow (DsRedKPP) dust and left overnight to recover. The following morning, between 7:00 and 8:00 h, the males were released into the relevant sub-enclosure within one of the semi-field cages (Fig. 1a). This contained a weighted mesh canopy drop-net (3 × 3 × 2 m) with a quick release drawstring positioned in the centre (Fig. 1b), fixed in the raised position as the males were released. Each sub-enclosure also contained a partially covered and water-filled black bucket (diameter: 30 cm; height: 40 cm) resting site (Fig. 1a). After one hour of acclimation, a single human host entered the sub-enclosure and walked around the perimeter once. They then positioned themselves under the raised drop-net for a period of 10 min. At this time, the drop-net was released capturing any males in close proximity to the host. Males trapped within the net were then collected using either a prokopack [75] or CDC vacuum aspirator. We also separately collected all males that were not trapped in the drop-net (i.e. those that were unresponsive to the host) with the use of vacuum aspirators and a BG-Sentinel trap, which was left running inside the sub-enclosure until 18:00 h.

Recaptured males were counted and the numbers collected from each treatment (KPPWT or DsRedKPP) determined by dust-colour verification. The proportion of males from each group that attended the swarm site was determined by dividing the number found within the drop-net by the total number recaptured. This assay was repeated with a new set of males three times in two replicates for each diet-water treatment (*n* = 6 trials per treatment). A total of 13 dusted males (from 480 released) were caught in traps placed outside the semi-field cage sub-enclosures (Fig. 1a). These individuals were not included in our analysis but confirmed that the escape-rate from the sub-enclosures was low (< 3%).

### Statistical analyses

All statistical tests were run using the R software environment [76] using a combination of packages *car* [77], *lsmeans* [78], *lme4* [79], *multcomp* [80], *dplyr* [81] and *coxme* [82]. To test whether larval diet-water treatment had an effect on adult male body size, we used an ANOVA model of wing length data with larval diet amount (high or low), rearing water type (laboratory or field), experimental replicate (1 or 2), and their interactions. All *post-hoc* comparisons were made using Tukey’s HSD test with a Bonferroni correction to account for multiple contrasts. We tested the effect of larval water source and diet amount on the abundance of energy reserves of newly emerged males using a general linear model (GLM). *Post-hoc* comparisons between treatment groups were made using Tukey’s HSD test with a Bonferroni correction. Mann-Whitney U-tests were then used to compare lipid, glycogen and sugar levels between the KPPWT and field-collected strains. We investigated the effect of larval diet treatment and adult diet on adult mortality using censored Cox proportional hazards models. *Post-hoc* pairwise comparisons between separate treatments were made using log-rank tests [83] with *P*-values adjusted where appropriate using the Bonferroni correction to account for multiple contrasts. To investigate the overall relative success of DsRedKPP males compared to the KPPWT line in small-cage mating competition experiments we used Pearson’s chi-square test. A mixed effects binary logistic regression model (GLMM) was used to explore the effect of larval diet-water condition on mating competition outcome (whether the successful male was from the DsRedKPP or KPPWT line). Larval treatment (HL, HF, LL or LF) was included as a fixed effect, while replicate and block were added as random effects.

In the semi-field cage mating competition assay, we used a weighted binomial mixed effects regression model (GLMM) to explore the effect of larval diet-water condition on DsRedKPP male mating success. The proportion of DsRedKPP-mated females, weighted by the total number of females collected, was modelled as a function of larval treatment (HL, HF, LL or LF), with replicate and semi-field cage identity included as random effects. The number of males attending swarm sites across the two mosquito strains used (DsRedKPP and wild-type) was assessed using a Kruskal-Wallis rank sum test. To investigate whether larval diet-water treatment had an effect on the proportion of males attending swarm sites, we used a weighted binomial mixed-effects model (GLMM). The proportion of DsRedKPP males caught inside the drop-net, weighted by the total recaptured, was modelled as a function of larval condition (HL, HF, LL or LF), with replicate included as a random effect. Differential recapture rates in the semi-field experiments were assessed using separate Poisson mixed effects models. Either the total number of females (mating competition) or males (swarm site attendance) collected were included as the response variable, with semi-field cage identity and larval treatment included as fixed effects, and replicate and experimental block as random effects. *Post-hoc* pairwise comparisons (across semi-field cages) were performed using Tukey’s HSD test with a *P*-value correction.

## Results

### Adult body size and nutrition

**Fig. 2:**
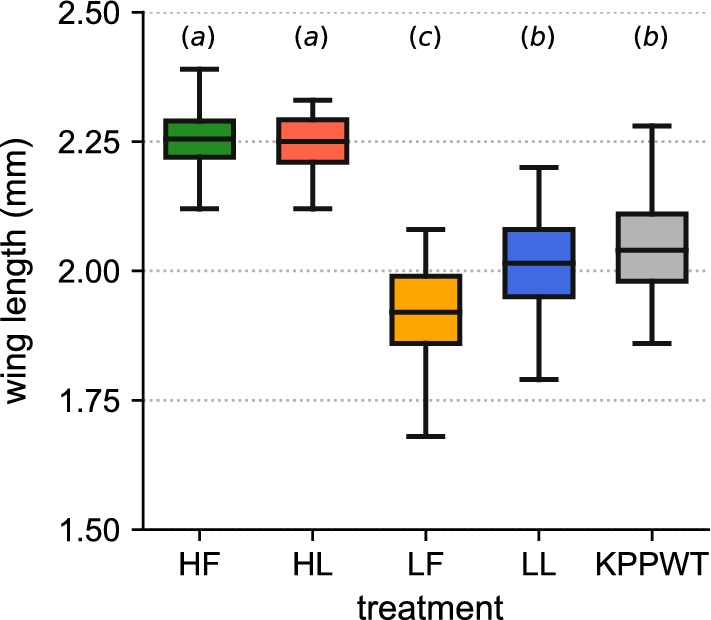
The distribution of adult male wing lengths for samples taken from DsRedKPP and KPPWT strains, separated by larval treatment. Data are aggregated across all experimental blocks. Treatments with the same letter-label (*a*, *b*, *c*) were not significantly different from one another. Different letters indicate significant differences between respective treatments. Sample sizes for each group: HF (*n* = 76); HL (*n* = 68); LF (*n* = 77); LL (*n* = 72); KPPWT (*n* = 294)

Manipulation of rearing conditions led to differences in the adult body sizes and nutritional reserves of males from different larval treatments (Fig. 2, Additional file 2: Table S3, Additional file 3). The interaction between food amount and rearing water type significantly affected body size (ANOVA: *F*_(1, 578)_ = 25.20, *P* < 0.0001). High diet treatments produced males that were larger than low or wild-type conditions, but which were not significantly different from one another. At low larval diets, individuals reared in field-container water were found to be significantly smaller than those from laboratory water, which themselves were not different in size from the KPPWT strain. Furthermore, we detected a significant replicate effect within treatments (ANOVA: *F*_(1, 578)_ = 12.88, *P* < 0.0001), which was found to affect males from the LF and KPPWT conditions only (Additional file 2: Figure S2). This did not, however, influence the overall size-trend between treatments across blocks.

Males from the high diet treatments were found to have a greater lipid content than those from low diet treatments (GLM: *F*_(2, 43)_ = 32.59, *P* < 0.0001), but did not differ in terms of their glycogen or sugar reserves. Rearing water source had no significant effect on nutritional abundance (GLM; lipids: *F*_(1, 28)_ = 0.17, *P* = 0.69; glycogen: *F*_(1, 47)_ = 2.22, *P* = 0.14; sugar: *F*_(1, 46)_ = 0.17, *P* = 0.69). The KPPWT males had a nutritional profile similar to those from the DsRedKPP LL and LF treatments (Additional file 2: Table S3), with a significantly lower stored lipid amount than the HF treatment group (Additional file 2: Table S3, Tukey’s HSD test; *P* < 0.0001), and marginally significantly less than HL (Additional file 2: Table S3, Tukey’s HSD test; *P* = 0.06). When compared to field-collected males, those from the KPPWT strain had similar lipid stores (54.77 ± 18.48 *vs* 59.10 ± 8.70 mg; Mann-Whitney U-test; *U* = 29.50, *n* = 16, *P* = 0.52), but a significantly higher glycogen (66.78 ± 22.00 *vs* 34.64 ± 27.99 mg; Mann-Whitney U-test; *U* = 94.00, *n* = 21, *P* = 3.00 × 10^−3^) and sugar (31.44 ± 12.09 *vs* 18.64 ± 7.79 μg, Mann-Whitney U-test; *U* = 88.50, *n* = 21, *P* = 0.012) content.

### Adult survival assay

**Fig. 3:**
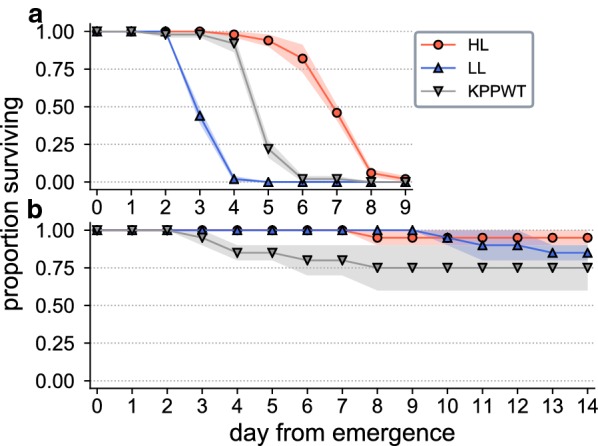
Adult male survival of individuals from different larval treatments offered a diet of either water (**a**) or 10% sugar solution (**b**). Data plotted show the mean across all replicates (*n* = 5 per treatment for water; *n* = 2 per treatment for sugar), with shaded regions indicating the standard error

As expected, overall mortality rates (Additional file 3) were higher for males offered a water-only diet (Fig. 3a) in comparison to those held on 10% sugar solution (Fig. 3b). Larval diet amount was found to be a significant factor in determining individual survival probabilities when adult males were sustained on both the water-only (Cox Proportional Hazards model log-rank test; *χ*^2^_(2)_ = 186.25, *P* = 3.78 × 10^−41^) and sugar-based (Cox Proportional Hazards model log-rank test; *χ*^2^_(2)_ = 6.25, *P* = 4.39 × 10^−2^) diets. When sustained on water only, males from the high-diet (HL) condition had a significantly greater survival likelihood than those from the wild-type strain (KPPWT), which in turn survived significantly longer than low-diet (LL) individuals (*post-hoc* pairwise tests with Bonferroni correction; *P* < 0.05). There were no significant mortality differences between larval treatments on the sugar-based diet (*post-hoc* pairwise tests with Bonferroni correction; *P* > 0.05).

### Laboratory mating competition

**Fig. 4:**
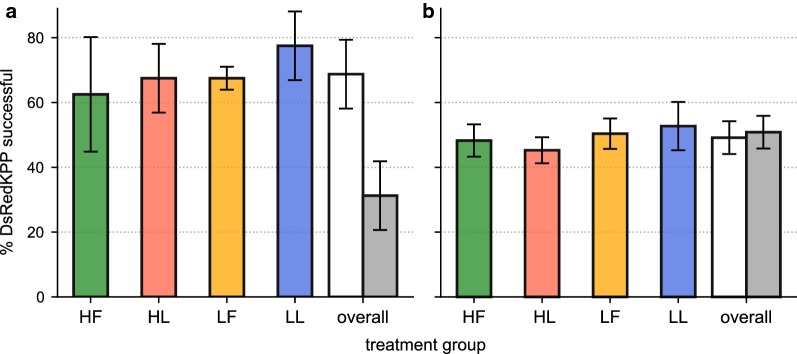
Results from the small cage and semi-field mating experiments. **a** The mean (± standard deviation) proportion of trials in which a DsRedKPP male was the first to copulate with a KPPWT female in small cage mating competition assays. **b** The mean (± standard deviation) proportion of mated recaptured females that were found to be inseminated by DsRed-tagged sperm in semi-field mating assays. Data are averaged across each replicate and separated by larval treatment. For each graph, the “overall” treatment group shows the DsRedKPP success rate averaged across all treatments (white bars) *versus* the corresponding average KPPWT success rate (grey bars)

DsRedKPP males were more likely to form a copula with a virgin wild-type female in laboratory assays (Fig. 4a, Pearson’s chi-square test; *χ*^2^_(1)_ = 22.50, *P* = 2.10 × 10^−6^) and were successful in 68.8% of matings when in competition with the KPPWT strain (Additional file 3). The specific diet-water treatment that larvae were reared on was not, however, found to affect relative mating success between the groups (GLMM; *P* > 0.05 for all treatment levels).

### Semi-field cage recapture rates

We investigated individual recapture rates in semi-field assays to determine potential sources of bias within the data collected. During semi-field mating competition experiments, female recapture rates were significantly affected by the semi-field cage used (Table 1, type-III ANOVA run on GLMM; Wald *χ*^2^ = 9.53, *P* = 8.54 × 10^−3^). Recapture rates were significantly greater in cage 2 than either cage 1 or 3 (Table 1). This pattern was weakly mirrored in the swarm site attendance tests (Table 1). However, the number of males collected did not differ by strain (DsRedKPP *vs* KPPWT, Kruskal-Wallis test; *χ*^2^ = 0.30, *P* = 5.81 × 10^−1^), and neither larval treatment nor the particular semi-field cage used were found to significantly affect the total recapture count in this assay (type-III ANOVA run on GLMM; *P* > 0.50 for both factors). Given the low escape rates observed (< 3%), this suggests an overall mortality rate between 10% and 40% within the semi-field cages.

**Table 1:** Mean (± standard deviation) total number of released adult females and males that were recaptured in, respectively, the semi-field mating competition and swarm site attendance assays, separated by cage number. Data are calculated across all trials performed within the given semi-field cage

Cage	♀ recaptured, mating competition (%)	♂ recaptured, swarm site attendance (%)
1	53.5 ± 2.12	68.8 ± 11.88
2	81.0 ± 2.65*	76.9 ± 16.24
3	61.3 ± 7.09	70.0 ± 16.04

*Indicates significant difference with *P* < 0.0001 from pairwise Tukey’s HSD tests

### Semi-field cage mating competition

The proportion of recaptured females that were found to be mated was consistently high (> 92%, Additional file 2: Table S4, Additional file 3). Of these, an average of 49.2% were identified as having been inseminated by males from the DsRedKPP strain (Fig. 4b, Additional file 2: Table S4). Larval condition was not found to significantly affect male mating success in these assays (GLMM; *P* > 0.50 for all conditions).

### Swarm site attendance

**Fig. 5:**
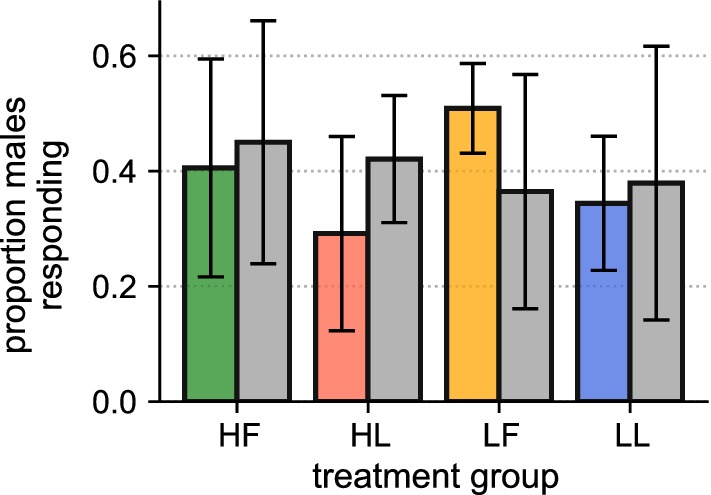
Swarm site attendance in semi-field cage sub-enclosures for DsRedKPP (coloured bars) and KPPWT (grey bars) strains. Bars show the mean (± standard deviation) proportion of all recaptured males that were collected inside the drop-net, separated by larval condition. All data shown are averaged over 6 trials per treatment

The mean number of males collected inside the drop-net was 5.63 ± 1.95, giving an average swarm site attendance rate of 39.53 ± 11.28% (Additional file 3). The number of males attending swarm sites did not vary by mosquito strain (DsRedKPP *vs* wild-type, Kruskal-Wallis rank-sum test; *P* > 0.50). Furthermore, when variation due to replicate effects was controlled for, swarm site attendance did not differ by diet-water treatment (Fig. 5, type-III ANOVA run on GLMM; *P* > 0.10 for all treatment levels).

## Discussion

The mating success of mosquito lines intended for release is a key consideration for the design of population control programs intended to reduce disease transmission. Importantly, the ability of transgenic males or those infected with *Wolbachia* or other sterilizing/transmission blocking bacteria to locate and compete for wild conspecifics in natural habitats is a fundamental aspect of their fitness [10]. While larval condition is known to influence various life history traits in mosquitoes, its effect on adult mating performance is not fully understood.

Here, we used a combination of laboratory and semi-field assays to investigate the joint effects of larval nutrition and aquatic environment on the mating success of a genetically modified strain of *Ae. aegypti* in direct competition with a wild-type line sourced from Kamphaeng Phet (KPP), Thailand. Variation in larval rearing condition did not influence male mating success across the treatments used. Neither increasing the amount of food available to larvae nor supplementing their aquatic habitat with field-container water improved performance relative to the wild-type strain. However, we observed a large discrepancy between laboratory and field-based measures of mating fitness, with transgenic males performing significantly better than wild-types in laboratory assays, an advantage that was not observed in the semi-field system. These findings suggest that differences in experimental designs can alter the perceived quality of lines if they are only measured under particular conditions. It is thus possible that mosquito strains intended for use in control programs, whose genetic, behavioural, and rearing backgrounds (including the potential effects of adaptation to laboratory environments [54, 55]) are often quite different to members of the target population into which they are released, could have a higher estimated performance ability if they are only assessed in laboratory settings. Importantly, our results highlight the need to incorporate field-based measurements of mating performance as part of control evaluations.

Variation in adult male body size between larval treatment groups was predominantly driven by food quantity (Fig. 2) [46]. Cohorts fed lower diet amounts had a smaller average body size, but greater size variance, than those in the high-diet groups, which yielded males of similar proportions (Fig. 2). Reduced food quantity results in staggered emergence of adult *Ae. aegypti* [84] and may alter underlying physiological mechanisms of development that are dependent on environmental factors (such as temperature [22]). Climatic conditions varied drastically over the rearing period. The interaction of nutritional level with other controls on larval growth may account for our observation of significant differences in the body sizes of males reared on low level diets across experimental blocks. It is also possible that other determinants of mosquito development, such as the structure of the gut microbiota [29], interact with food quantity to influence adult morphology. Mosquitoes from different rearing environments harbour distinct bacterial communities [30], and those raised under laboratory protocols are likely dissimilar to natural field populations [85]. Interestingly, we note that only lower diet, field-container water treatments (LF and KPPWT) displayed a significant size-replicate effect, indicative of a high variation between rearing cohorts that was not present in high diet and laboratory water conditions (Additional file 2: Figure S2). The interactions between microbiome, nutrition, and environment in determining adult mosquito physiology are likely both highly sensitive and complex, and further work is required to understand their relative roles in developmental processes.

Our findings support the notion that male mating performance in *Aedes* is more nuanced than “bigger is better” [45], as appears to be the case for *Anopheles* mosquitoes [86, 87]. Recent analyses of mating in *Ae. aegypti* have indicated that success may be influenced by a relative size-assortative relationship [44, 45]. Size-assortative mating could be driven by the mate-grasping mechanics of *Aedes* copulation, which requires males to seize and orient venter-to-venter with females using their legs in order to facilitate genital contact [88, 89]. As a result, it has been suggested that release lines may enjoy greater mating success if their body size distribution more closely matches that of the target field group [44, 45]. While our results did not reveal a significant effect of larval diet quantity (and therefore body size, Fig. 2) on male mating competitiveness, we note that DsRedKPP males reared on a low larval diet quantity (which had a larger size overlap with the competitor KPPWT line) were observed to perform marginally better in mating assays than those from high diet conditions. Establishing the strength of this effect, and how it may contribute towards the potential performance of release lines in the field, is a worthwhile avenue for future investigation.

During laboratory-based small-cage mating trials, males from the DsRedKPP line were consistently more successful in being the first to form a copula with a KPPWT female, no matter what their larval condition (Fig. 4a). This is potentially a consequence of adaptation of the DsRedKPP strain to mating in such confined enclosures during colonisation and laboratory maintenance (see Additional file 1: Text S1). Competitive selective pressures are thought to be high in such rearing environments. Recent experimental evidence indicates that evolutionary responses in mating performance can emerge in as little as five generations in this species [90]. Practically, this would confer a greater advantage to the DsRedKPP males during laboratory mating competition assays, which may explain the discrepancy between the measures of sexual fitness that we observed. However, it is important to note that differences between the mating performance of the DsRedKPP and KPPWT lines may also be attributable to variation in other genetic and rearing factors between the strains. Establishing the effects of laboratory adaptation on mosquito performance is currently an active field of investigation [54], and further work is required to understand its implications for mating fitness in mass-produced lines.

In the semi-field assays, DsRedKPP males performed comparably to KPPWT individuals no matter which larval treatment they came from. Males from the laboratory strain showed similar levels of swarm site attendance (a proxy for their attractiveness towards mating arenas, Fig. 5) and achieved a comparable rate of female insemination in large-scale mating trials (Fig. 4b) when compared to their wild-type counterparts. While we were not able to detect specific instances of multiple female insemination (mating of a single female by both a DsRedKPP and KPPWT male), rates of polyandry in semi-field colonies of *Ae. aegypti* are known to be low [91], and unlikely to substantially alter the results presented here. Furthermore, and in contrast to observations from previous investigations of laboratory mosquito strains [92], the males used in this study appeared to respond appropriately to swarming cues (Fig. 5). Under natural conditions, therefore, there appears to be little impact of laboratory habituation or genetic modification on male mating competitiveness, a finding that corroborates reports elsewhere from studies of *Aedes* mosquitoes [56, 58, 59, 61, 93–95]. The DsRedKPP strain used here originated from a cross between a long-term laboratory colony and a low generation line (see Additional file 1: Text S1). There is evidence that reproductive phenotypes in laboratory-adapted mosquito strains can be restored, at least partially, to wild-type levels by crossing between lines [63]. Our results suggest that where laboratory-induced habituation costs on mating behaviours do exist, they can potentially be mitigated through colony-hybridization. The relative benefits of outbreeding transgenic strains with laboratory [63] and field [53] material on mating performance warrants deeper consideration.

Standardised assessments are now recognised as a requirement to determine the quality of release strains prior to deployment in the field [14, 96–98]. While in this study we used a model transgenic system not intended for use as part of any mass-release program, our results nevertheless demonstrate that different approaches to measuring mating success can produce quite different outcomes. These findings highlight the importance of testing different fitness measures across a range of environments; success in laboratory settings does not necessarily translate to high performance in natural scenarios.

## Conclusions

Our findings show little effect of larval diet and rearing environment on adult sexual competitiveness of a transgenic line when compared to wild-type counterparts, but offer several important insights for the development and testing of mass-reared lines. First, we highlight the discrepancy between laboratory-based and field-based metrics of mating success. Laboratory assays may present conditions advantageous to habituated strains, which taken alone, could lead to an overestimation of their performance in field situations. We stress the importance of validation between approaches used to assess the quality of different release lines; further work is required to determine specific fitness effects on varied transgenic systems developed for use in unique mosquito control programs. Secondly, it has been suggested that optimising the fitness of release strains may fundamentally rely on a trade-off between characteristics. For instance, selecting for larger males, which our results show to be positively correlated with longevity (Fig. 3) [28], and which has previously been associated with increased sperm transfer [25, 26], could improve male opportunities to mate in low density field environments [99]. However, previous work indicates that this may come at a cost of reduced sexual competitiveness due to a size-mismatch with the target population [44, 45]. Our results do not support the existence of a strong size-assortative mating effect in semi-field mating assays, yet further work is required to fully explore the potential implications of body size on release line fitness in natural conditions. Finally, while we do not find clear evidence for an influence of larval condition on adult male mating success, we cannot rule out the possibility that diet and rearing water source may impact field performance after long-term selection. Recommendations to maintain the fitness and mating competitiveness of laboratory insect strains destined for controlled release include diet variation and supplementation, and frequent backcrossing with wild-types to promote an outbred genetic background [53]. Ultimately, this requires both a detailed knowledge of developmental and physiological characteristics of the target population, coupled with a better understanding of mosquito mating biology. Taken together, these efforts have the potential to drastically improve mosquito control technologies.

## Additional files

**Additional file 1: Text S1.** Supplementary methods detailing generation of the DsRedKPP strain. **Text S2.** Determination of high and low laboratory diet quantities *via* a preliminary pilot study. **Figure S1.** Weighted larval development and pupation rates across pilot diet treatments, plotted as a function of time (from hatch). **a** Mean daily instar (± standard error) of all remaining larvae (i.e. those which had not pupated or died). **b** Total number of pupae counted each day. **Table S1.** Wing lengths of adult males from different diet treatments (*n* = 12 per treatment).

**Additional file 2: Table S2.** Summary of experimental blocking design. **Table S3.** Nutritional content analyses of mosquitoes in experimental treatment groups. **Figure S2.** Wing lengths of adults from all larval treatments, separated by experimental block. **Table S4.** Full results of semi-field cage mating competition experiment.

**Additional file 3: Dataset S1.** Supporting data from wing length measurements, daily adult survival assay, laboratory mating competition experiments, semi-field cage mating competition experiments, and semi-field cage swarm site attendance experiments.

## Data Availability

The datasets supporting the conclusions of this article are included within the article and its additional files.
