# Supplementary material for: Too “sexy” for the field? Paired measures of laboratory and semi-field performance highlight variability in the apparent mating fitness of Aedes aegypti transgenic strains
Source: Parasit Vectors. 2019 Jul 19;12:357. doi: 10.1186/s13071-019-3617-2 (PMC6642483; doi:10.1186/s13071-019-3617-2)
Supplement: Supplementary file 1 — Additional file 1: Text S1. Supplementary methods detailing generation of the DsRedKPP strain. Text S2. Determination of high and low laboratory diet quantities via a preliminary pilot study. Figure S1. Weighted larval development and pupation rates across pilot diet treatments, plotted as a function of time (from hatch). a Mean daily instar (± standard error) of all remaining larvae (i.e. those which had not pupated or died). b Total number of pupae counted each day. Table S1. Wing lengths of adult males from different diet treatments (n = 12 per treatment). [file 13071_2019_3617_MOESM1_ESM.pdf]

Additional file 1: Too "sexy" for the field? Paired measures of laboratory and semi-field performance highlight variability in the apparent mating fitness of *Aedes aegypti* transgenic strains

Andrew Aldersley, Arissara Pongsiri, Kamonchanok Bunmee, Udom Kijchalao, Wachiraphan Chittham, Thanyalak Fansiri, Nattaphol Pathawong, Alima Qureshi, Laura C. Harrington, Alongkot Ponlawat, Lauren J. Cator

Text S1: Supplementary methods detailing generation of the DsRedKPP line

A laboratory strain of *Aedes aegypti* Higgs' White Eye (HWE) DsRed mosquitoes [1] was crossed with a low-generation KPP field line to produce the DsRedKPP strain used in experimental assays. This process involved several stages, briefly outlined here. Firstly, a batch of ~500 HWE DsRed and KPP (2nd and 3rd generation from field) mosquitoes were reared using the general approach outlined in the main text (larvae were fed 0.5mg/larvae/day ground fish food), with adults placed in sex-separated cages and held on 10% sucrose solution. Virgin males from each line were then mixed with virgin females from the opposite strain and allowed to mate. Females were offered a blood meal of defibrinated horse blood every 2-3 days and encouraged to oviposit. Next, we hatched 1000 eggs from the initial cross (which generated offspring heterozygous for the DsRed trait), using the same rearing conditions described above. Adults from this cross were allowed to freely mate. Females were bloodfed and allowed to oviposit; the resultant offspring contained a mixture of individuals, some homozygous for the DsRed trait.

For the third stage, 1000 eggs from the second cross were hatched and reared under standard conditions. Pupae were screened for DsRed fluorescence using a stereomicroscope (ZEISS SteREO Discovery.V8) with a DsRed filter attachment. Note that the DsRed indicator is only visible in male pupae. We collected 150 DsRed-positive pupae and allow them to eclose in a cage. 100 adult males were then selected from this this population subset, ensuring only those with black eyes (i.e. non-HWE) were chosen. The remaining pupae were placed individually in separate 15mL falcon tubes and allowed to eclose. 100 females (also non-HWE) were obtained and paired individually with a single DsRed-positive male in a small container (0.45L). Each pair was provided with a 10% sucrose solution and left for 48 hours. After this time, females were extracted and placed into individual numbered oviposition tubes, offered a

blood meal, and allowed to oviposit. Egg papers collected from each female were numerically tagged such that they could be identified. We collected at least 25 eggs from each female (>2500 eggs total).

For the final stage, eggs collected from the one-on-one crossings were hatched and reared separately in small containers and allowed to pupate. Pupae were screened for DsRed fluorescence; only those from DsRed-producing females were retained and allowed to eclose. These individuals were placed into a cage and allowed to freely mate, generating offspring homozygous for the DsRed marker. Females were continually offered a blood meal every 2-3 days. We collected approximately 10000 eggs from this cross (the DsRedKPP line) which were subsequently used in experimental assays.

Text S2: Determination of high and low laboratory diet treatment conditions

We conducted a pilot study to determine the food quantities used in the “high” and “low” laboratory diet treatment conditions in experimental assays. Three 1st instar DsRedKPP larvae were placed into 3mL of deionised water in a single well of a 6-well tissue culture plate and fed on one of 5 laboratory diet regimes, equivalent to 0.1, 0.3, 0.5, 0.7, or 1.0mg/larvae/day (ground fish food, Cichlid Gold, Hikari, Kyrin Food Industries Ltd, Japan). The diet concentrations were ground up in water to make a “slurry” of known concentration that was fed to the larvae by pipette in the appropriate volume. We ran 5 plates per diet concentration (so 30 wells per food amount, 90 mosquitoes per concentration). Containers were held in a climate-controlled laboratory at 30°C, 70% relative humidity, to approximate anticipated field conditions. Larvae were fed daily using the food amounts prescribed above. Wells were monitored each day for mortality and larval development stage (i.e. the number of larvae at each instar). Larval instars were determined by counting larval molt. Pupae were allowed to eclose and the right wings of 12 adult males from each treatment were removed to estimate body size differences across groups. These data was used to identify the early differences in larval development and mortality that are correlated with significant differences in adult body size.

On average, larvae developed at approximately the same rates across all diet treatments, with the exception of the lowest food amount, in which larval development was slower than the other four diet conditions (Figure S1a). As expected, mortality was also greatest amongst

larvae on the 0.1mg/day diet, although overall survival rates were relatively high (>80%) in all treatments over the period of the experiment. The higher diet treatment groups (0.7 and 1.0 mg/larva/day) showed peaks in the number of 4th instar larvae and pupae on days 3 and 4, respectively, 24 hours earlier than in the intermediate diet groups (0.3 and 0.5 mg/larva/day, Figure S1b). The intermediate diet groups spent longer at the 4th instar stage, but this was the only real difference observed at this point. Pupal development tended to be more "peaked" around a window of a couple of days in the high diet groups, whereas it followed a broader distribution in the intermediate groups.

Figure S1: Weighted larval development and pupation rates across pilot diet treatments, plotted as a function of time (from hatch). (a) Mean daily instar (± standard error) of all remaining larvae (i.e. those which had not pupated or died). (b) Total number of pupae counted each day.

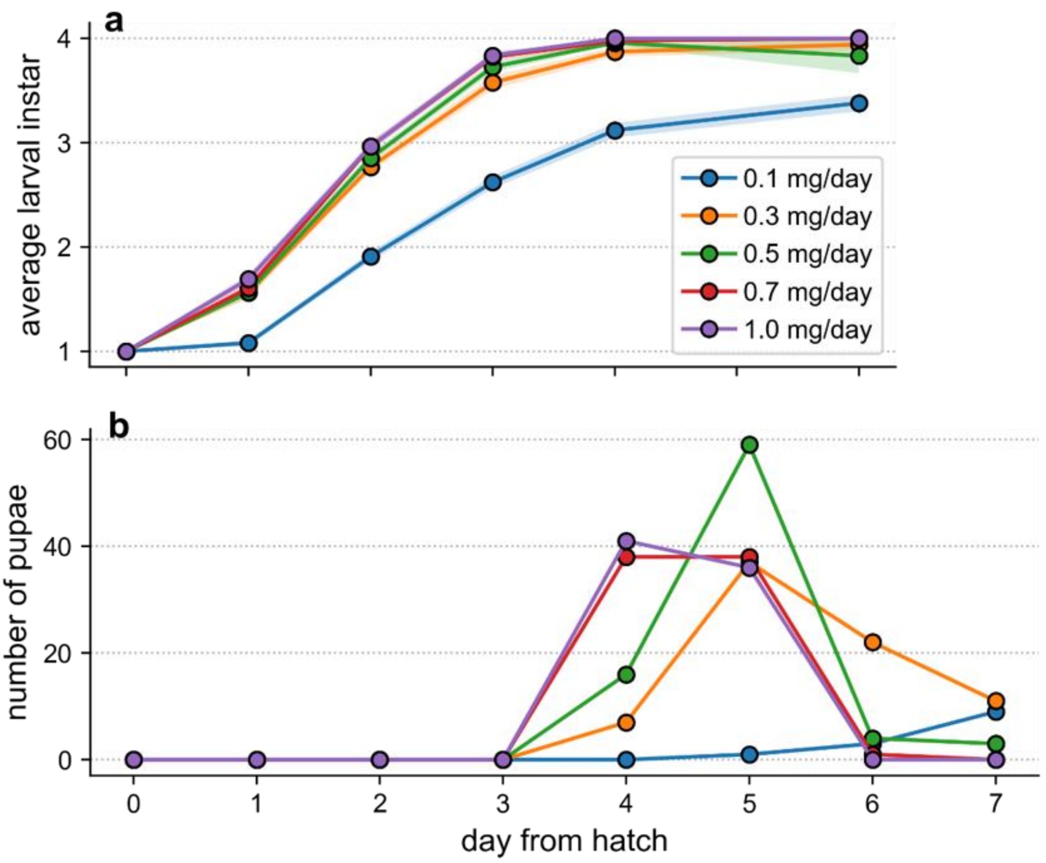

Adult male wing lengths varied significantly across diet treatments (Table S1, one-way ANOVA; $F = 31.18, p = 2.33 \times 10^{-13}$). Tukey post-hoc analyses indicated a significant difference between wing lengths of the lowest diet group and all others, as well as between the 0.3 and 1.0 mg/larva/day treatments, but no others. Using these results, it was decided that the

two diet treatment groups to be used in the field would be 0.3mg/larva/day (low amount) and 1.0mg/larva/day (high amount). These two treatments yielded adult males with significantly different physiology (Table S1), while offering similar developmental and survival rates, making their use practical in a field setting.

Table S1: Wing lengths of adult males from different diet treatments ($n = 12$ per treatment).

Diet (mg/larva/day)	Mean wing length (mm)	Standard deviation
0.1	1.76	0.14
0.3	2.05	0.09
0.5	2.07	0.09
0.7	2.14	0.09
1.0	2.17	0.06

References

1. Smith RC, Walter MF, Hice RH, O'Brochta DA, Atkinson PW. Testis-specific expression of the β2 tubulin promoter of *Aedes aegypti* and its application as a genetic sex-separation marker. Insect Mol Biol. 2007;16:61–71.
