# Supplementary material for: Too “sexy” for the field? Paired measures of laboratory and semi-field performance highlight variability in the apparent mating fitness of Aedes aegypti transgenic strains
Source: Parasit Vectors. 2019 Jul 19;12:357. doi: 10.1186/s13071-019-3617-2 (PMC6642483; doi:10.1186/s13071-019-3617-2)
Supplement: Supplementary file 2 — Additional file 2: Table S2. Summary of experimental blocking design. Table S3. Nutritional content analyses of mosquitoes in experimental treatment groups. Figure S2. Wing lengths of adults from all larval treatments, separated by experimental block. Table S4. Full results of semi-field cage mating competition experiment. [file 13071_2019_3617_MOESM2_ESM.pdf]

Additional file 2: Too "sexy" for the field? Paired measures of laboratory and semi-field performance highlight variability in the apparent mating fitness of *Aedes aegypti* transgenic strains

Andrew Aldersley, Arissara Pongsiri, Kamonchanok Bunmee, Udom Kijchalao, Wachiraphan Chittham, Thanyalak Fansiri, Nattaphol Pathawong, Alima Qureshi, Laura C. Harrington, Alongkot Ponlawat, Lauren J. Cator

Table S2: Summary of experimental blocking design.

Block	Treatments (replicate)
1	HL(1), HF(1), LL(1)
2	HL(2), LL(2), LF(1)
3	HF(2), LF(2)

Table S3: Nutritional content analysis for mosquitoes in the experimental treatment groups. Presented are the mean and standard deviation glycogen, lipid, and sugar abundance levels (µg/mosquito) in freshly eclosed males from each of the experimental treatment groups. Superscripts a, b, and c denote statistically significant differences from the GLM model described in the main text.

Treatment	Glycogen (µg)	Lipid (µg)	Sugar (µg)
HF	64.33 ± 12.52^{a}	114.90 ± 43.53^{a}	24.52 ± 7.19^{a}
HL	71.28 ± 10.83^{a}	98.45 ± 27.42^{ac}	28.79 ± 8.49^{a}
LF	58.77 ± 10.95^{a}	58.28 ± 7.23^{b}	28.84 ± 8.74^{a}
LL	66.56 ± 26.11^{a}	57.49 ± 14.87^{b}	26.72 ± 12.82^{a}
KPPWT	66.76 ± 22.00^{a}	59.10 ± 8.70^{bc}	31.44 ± 12.09^{a}

Figure S2: Wing lengths of adult males from all larval treatments, separated by experimental block.

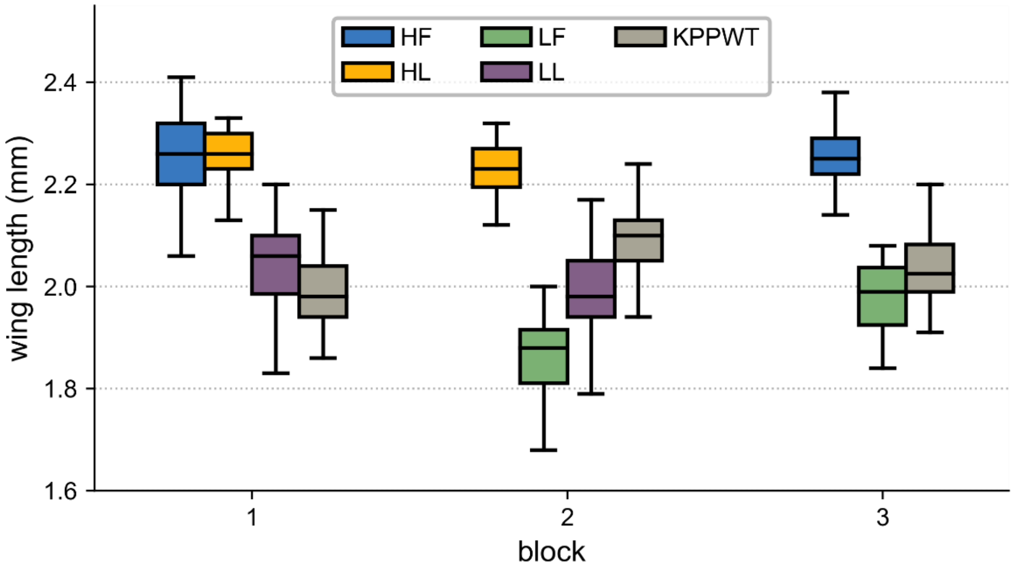

Table S4: Results of semi-field cage mating competition experiments, separated by larval treatment and replicate. Data show the number of females recaptured, the proportion of recaptured females that were mated, and the proportion of mated females that were inseminated with DsRed-tagged sperm.

Treatment	# females recaptured		% females mated		% mated by DsRedKPP		
	Rep 1	*Rep 2*	*Rep 1*	*Rep 2*	*Rep 1*	*Rep 2*	*Mean*
HF	60	78	93.3	97.4	51.8	44.7	48.3
HL	83	69	95.2	95.7	48.1	42.4	45.3
LF	55	55	92.7	98.2	47.1	53.7	50.4
LL	52	82	96.2	95.1	58.0	47.4	52.7
